# The association between obesity and glaucoma in older adults: evidence from the China Health and Retirement Longitudinal Study

**DOI:** 10.4178/epih.e2023034

**Published:** 2023-03-09

**Authors:** Xiaohuan Zhao, Qiyu Bo, Junran Sun, Jieqiong Chen, Tong Li, Xiaoxu Huang, Minwen Zhou, Jing Wang, Wenjia Liu, Xiaodong Sun

**Affiliations:** 1 Department of Ophthalmology, Shanghai General Hospital (Shanghai First People’s Hospital), Shanghai Jiao Tong University School of Medicine, Shanghai, China; 2 National Clinical Research Center for Eye Diseases, Shanghai, China; 3 Shanghai Key Laboratory of Fundus Diseases, Shanghai, China

**Keywords:** Obesity, Glaucoma, Body mass index, China Health and Retirement Longitudinal Study

## Abstract

**OBJECTIVES:**

This study evaluated the association between obesity and glaucoma in middle-aged and older people. A population-based retrospective cohort study was conducted using data from the China Health and Retirement Longitudinal Study.

**METHODS:**

Glaucoma was assessed via self-reports. Multivariate logistic regression analysis and a Cox proportional hazards model were used to assess the relationship between obesity and glaucoma risk.

**RESULTS:**

Older males living in urban areas who were single, smokers, and non-drinkers were found to have a significantly higher incidence of glaucoma (all p<0.05). Diabetes, hypertension, and kidney disease were also associated with higher glaucoma risk, while dyslipidemia was associated with lower risk (all p<0.05). After the model was adjusted for demographic, socioeconomic, and health-related variables, obesity was significantly associated with a 10.2% decrease in glaucoma risk according to the Cox proportional hazards model (hazard ratio, 0.90; 95% confidence interval [CI], 0.83 to 0.97) and an 11.8% risk reduction in the multivariate logistic regression analysis (odds ratio, 0.88; 95% CI, 0.80 to 0.97). A further subgroup analysis showed that obesity was associated with a reduced risk of glaucoma in people living in rural areas, in smokers, and in those with kidney disease (all p<0.05). Obesity also reduced glaucoma risk in people with diabetes, hypertension, or dyslipidemia more than in healthy controls (all p<0.05).

**CONCLUSIONS:**

This cohort study suggests that obesity was associated with a reduced risk of glaucoma, especially in rural residents, smokers, and people with kidney disease. Obesity exerted a stronger protective effect in people with diabetes, hypertension, or dyslipidemia than in healthy people.

## INTRODUCTION

Glaucoma is a neurodegenerative disease and the leading cause of irreversible vision loss worldwide [1]. The global prevalence of glaucoma is estimated to be 3.5% among people aged 40 years to 80 years [2]. As the global population ages, it is predicted that 111.8 million people will have glaucoma in 2040 [2]. Since glaucomatous damage to the visual system is irreversible and requires lifelong treatment, glaucoma poses a substantial social burden. The diagnosis of glaucoma is frequently delayed as it may be asymptomatic until a relatively late stage. Thus, there is a need to identify protective and risk factors for glaucoma.

Glaucoma is a multifactorial disease, and the impact of obesity on glaucoma risk remains uncertain. Some studies have found associations between glaucoma and obesity or high body mass index (BMI) [4-6]. A cross-sectional study using data from the Korea National Health and Nutrition Examination Survey (KNHANES) found that not being overweight was significantly associated with primary open-angle glaucoma (POAG) [3]. Another study of a Korean population found that low BMI was only associated with a decreased risk of glaucoma in females and in subjects aged 40 years to 49 years [4]. However, another study of the same Korean population found that BMI was associated with POAG only in male participants [5]. A prospective cohort study using data from the Nurses’ Health Study found no significant relationship between BMI and POAG [6]. Additional large-scale cohort studies are needed to clarify the association between obesity and glaucoma.

**Figure 1.**
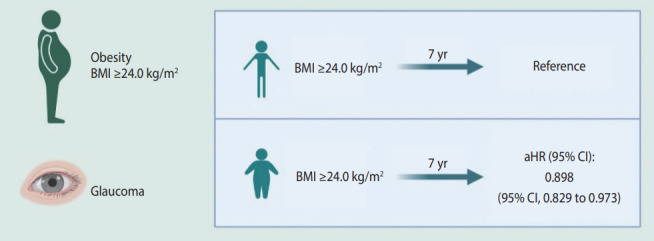
Association between obesity and glaucoma in older adults based on the China Health and Retirement Longitudinal Study. BMI, body mass index; aHR, adjusted hazard ratio; CI, confidencece interval.

The present retrospective cohort study explored the association between obesity and glaucoma after adjusting for socio-demographic characteristics and certain health conditions; the study used data from the China Health and Retirement Longitudinal Survey (CHARLS; Figure 1).

## MATERIALS AND METHODS

### Study population and design

This study analyzed data from CHARLS, a nationally representative longitudinal survey of people in China aged 45 years or older and their spouses [7]. In total, 150 county-level units from 28 provinces were randomly selected from a sampling framework that included all county-level units except Tibet. All sampling was computerized to avoid human manipulation. The baseline survey was conducted between June 2011 and March 2012; every 2 years thereafter, face-to-face computer-assisted personal interviews (CAPIs) were conducted with selected participants in 2013, 2015, and 2018, respectively. In the baseline study, 10,069 of the 17,708 randomly selected CAPIs participants were primary respondents, and 7,639 were spouses of primary respondents. CHARLS data include social, economic, and health data. Fasting blood samples were taken by trained nurses at township hospitals or at local offices of the Chinese Center for Disease Control and Prevention (CDC). All blood samples were shipped to the CDC in Beijing, where they were stored and analyzed. CHARLS was conducted by the National School of Development in China (China Center for Economic Research) at Peking University; the data are available at http://charls.pku.edu.cn/.

Multiple imputation was used to impute missing data. Baseline (2011) participants with missing data for age, sex, or obesity were excluded from our study. Participants who did not complete a glaucoma test or who were diagnosed with glaucoma prior to baseline data collection were also excluded. We also excluded participants for whom residence, education, or disease data (diabetes, dyslipidemia, or kidney disease) were missing. In total, 13,357 participants were included in our study; of these, 13,231, 12,842, and 11,868 received follow-up glaucoma assessments in 2013, 2015, and 2018, respectively.

### Body mass index and obesity assessment

BMI was used to measure overweight status and obesity. BMI was calculated as weight (in kilograms) divided by height (in meters). Participants’ weights and heights at baseline were measured by trained staff. Weight was measured to the nearest 0.1 kg without shoes, and height was measured to 0.1 cm.

Participants were assigned to the following quartiles based on baseline BMI: quartile 1 (<20.8 kg/m^2^), quartile 2 (20.8-23.1 kg/m^2^), quartile 3 (23.1-25.7 kg/m^2^), and quartile 4 (> 25.7 kg/m^2^). The World Health Organization definition of obesity (BMI>30.0 kg/m^2^) is not suitable for Asian populations due to skeletal and body shape differences. In this study, obesity was defined as a BMI of 24.0 kg/m^2^ or higher, in line with the recommendation for Chinese adults [9].

### Glaucoma assessment

Glaucoma was assessed via self-reports both at baseline and during follow-up. Participants were asked, “Has a doctor, nurse, or paramedical caregiver ever treated you for glaucoma?” Participants who responded “yes” were determined to have glaucoma. Participants with a previous glaucoma diagnosis at baseline were excluded from the present study, and the enrolled participants received follow-up glaucoma assessments in 2013, 2015, and 2018.

### Covariates

Demographic and socioeconomic data collected included age, sex, educational and marital status, and residential area. Educational level was categorized as primary or below, middle school, high school, or college or above. Residential area was categorized as urban or rural. Health behaviors included smoking and drinking. Smokers were categorized as current/former smokers or non-smokers. Drinkers were categorized as those who drank more than once a month, those who drank once a month or less, and nondrinkers. Data were also collected on diabetes, hypertension, dyslipidemia, and kidney disease. Diabetes was defined as fasting plasma glucose ≥ 7.0 mmol/L, glycated hemoglobin ≥ 6.5%, or a self-reported previous diagnosis of diabetes. Hypertension was defined as blood pressure ≥ 140/90 mmHg or self-reported use of antihypertensive medication. Dyslipidemia was defined as total cholesterol ≥ 6.2 mmol/L, low-density lipoprotein cholesterol ≥ 4.1 mmol/L, triglycerides ≥ 2.3 mmol/L, or high-density lipoprotein cholesterol < 1.0 mmol/L. Kidney disease was determined by a self-reported previous diagnosis or self-reported treatment for kidney disease or related complications.

### Statistical analysis

For baseline characteristics, the mean and standard deviation (SD) were calculated for continuous variables, and the percentage was calculated for categorical variables. The BMI quartiles were compared using one-way analysis of variance for continuous variables and the chi-square test for categorical variables. Multivariate logistic regression analysis and a Cox proportional hazards model were used to evaluate the association between BMI quartile and glaucoma; these associations were measured using odds ratios (ORs) or hazard ratios (HRs) with 95% confidence intervals (CIs). The multivariate logistic regression analysis included the variables that showed statistically significant results in the univariate analysis or clinically significant associations with glaucoma. Among them, the variables that showed statistically significant associations with glaucoma in the univariate analysis included sex, age, marital status, residential area, smoking, drinking, diabetes, hypertension and dyslipidemia; the variables that may be clinically significant included educational level and kidney disease. Model 1 was adjusted for age, sex, and obesity, and model 2 was further adjusted for education, area of residence, marital status, smoking, drinking, diabetes, hypertension, dyslipidemia, and kidney disease in the Cox proportional hazards model.

To investigate the long-term effects of obesity on glaucoma, we used a mixed-effects model to analyze the association of obesity at baseline with repeated follow-up glaucoma assessments. The main effect of obesity and visit time, together with the interaction term, were fitted into the mixed model. A mixed-effects model is more effective for handling missing data, as missing values have less impact on parameter estimates than in a general linear model. This analysis was adjusted for the same covariates used in model 2, and individual differences were treated as random-effects terms. We analyzed the effect of obesity stratified by age (< 50, 50-60, 60-70, and > 70 years), marital status (married/partnered or other), area of residence (urban or rural), smoking (smoker or non-smoker), diabetes (with or without diabetes), hypertension (with or without hypertension), dyslipidemia (with or without dyslipidemia), and kidney disease (with or without kidney disease). All analyses were conducted using SPSS version 23.0 (IBM Corp., Armonk, NY, USA).

### Ethics statement

Ethics approval for the data collection in the CHARLS was obtained from the Biomedical Ethics Review Committee of Peking University (IRB00001052-11015). Informed consent was not applicable.

## RESULTS

### Population characteristics

A total of 13,357 participants were enrolled in our study. Participants’ demographic information and health conditions are shown in Table 1, divided by BMI quartile. Of our participants, 3,364 (25.2%) had a BMI over 25.7 kg/m^2^, while 3,345 (25.0%), 3,328 (24.9%), and 3,320 (24.9%) had BMIs of less than 20.8 kg/m^2^, 20.8-23.1 kg/m^2^, and 23.1-25.7 kg/m^2^, respectively. Participants in the highest BMI quartile (> 25.7 kg/m^2^) were more likely to be younger, female, more educated, married or partnered, non-smokers, non-drinkers, and urban residents. Individuals with higher BMIs were particularly more likely to have diabetes, hypertension, and/or dyslipidemia, while participants with low BMIs were more likely to have kidney disease.

### Association of obesity with glaucoma

Multivariate logistic regression analysis was used to explore the association between obesity and glaucoma (Table 2). Obesity was significantly associated with an 11.8% decrease in glaucoma risk compared to participants who were not overweight (OR, 0.88; 95% CI, 0.80 to 0.97). Male sex (OR, 1.24; 95% CI, 1.10 to 1.40), higher age (60-70 years: OR, 1.31, 95% CI, 1.14 to 1.50; > 70 years: OR, 3.25; 95% CI, 2.79 to 3.79), residence in an urban area (OR, 1.60; 95% CI, 1.46 to 1.75), smoking (OR, 1.16; 95% CI, 1.03 to 1.30), and not drinking (OR, 1.15; 95% CI, 1.03 to 1.23) were also positively associated with glaucoma incidence. Participants who were married or partnered had a lower risk of glaucoma than single participants (OR, 0.65; 95% CI, 0.58 to 0.74). Participants with diabetes (OR, 1.38; 95% CI, 1.23 to 1.56), hypertension (OR, 1.25; 95% CI, 1.14 to 1.37), or kidney disease (OR, 1.20; 95% CI, 1.01 to 1.41) had a significantly elevated risk of glaucoma. However, participants with dyslipidemia had a lower risk of glaucoma (OR, 0.79; 95% CI, 0.72 to 0.87).

A Cox proportional hazards model was used to further evaluate the association between obesity and glaucoma after adjustment for cofounders. Model 1 showed no significant association between obesity and glaucoma (Table 3). However, in the multivariate-adjusted model (Model 2), obesity was significantly associated with a 10% decrease in glaucoma risk (HR, 0.90; 95% CI, 0.83 to 0.97). Male participants had a 18% (model 1: HR, 1.18; 95% CI, 1.09 to 1.27) or 18% (model 2: HR, 1.18; 95% CI, 1.07 to 1.31) higher risk of glaucoma than female participants. In model 1, age was significantly associated with an increased risk of glaucoma, ranging from 38% for participants aged 60-70 years (HR, 1.38; 95% CI, 1.23 to 1.55) to 205% for those over 70 (HR, 3.05; 95% CI, 2.72 to 3.42), both compared to participants under 50. In model 2, age was also significantly associated with an increased risk of glaucoma, ranging from 24% for those aged 60-70 years (HR, 1.24; 95% CI, 1.09 to 1.40) to 143% for those over 70 (HR, 2.43; 95% CI, 2.13 to 2.76), both compared to participants under 50 years of age. Married or partnered individuals had a 27% lower risk of glaucoma (HR, 0.73; 95% CI, 0.66 to 0.80). In the multivariate model, urban residence (HR, 1.45; 95% CI, 1.34 to 1.56), smoking (HR, 1.15; 95% CI, 1.04 to 1.26) and abstinence from alcohol (HR, 1.12; 95% CI, 1.02 to 1.23) negatively impacted glaucoma incidence. Diabetes (HR, 1.28; 95% CI, 1.16 to 1.42) and hypertension (HR, 1.21; 95% CI, 1.12 to 1.30) had negative impacts on glaucoma incidence, while dyslipidemia (HR, 0.83; 95% CI, 0.76 to 0.90) had a positive impact in model 2.

### Long-term effects of obesity on glaucoma

**Figure 2.**
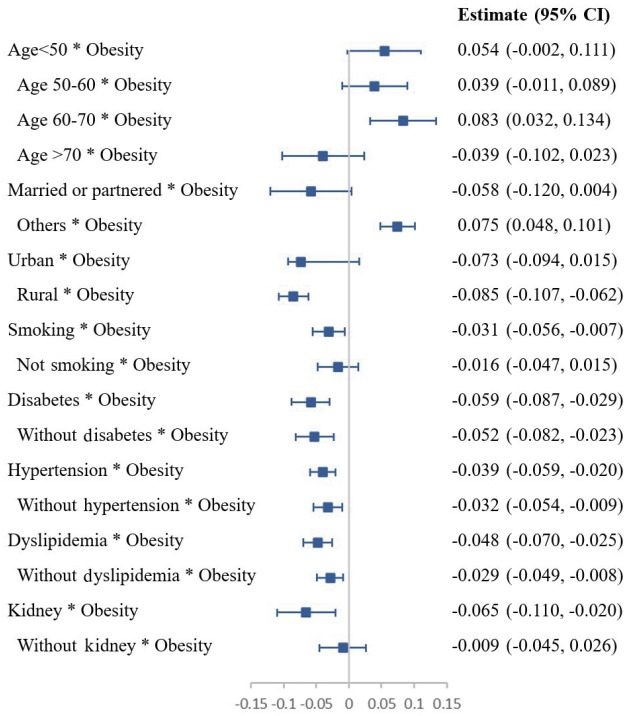
Longitudinal associations between obesity and glaucoma in subgroups. CI, confidence interval.

Glaucoma was not associated with obesity in model 1, but model 2 showed a significant association; this could have been due to interactions among independent variables. Therefore, we further analyzed the interaction effect of obesity and glaucoma using a linear mixed-effects model (Table 4). Of the 13,357 participants included in this study at baseline (2011), 13,231, 12,842, and 11,868 received follow-up glaucoma assessments in 2013, 2015, and 2018, respectively. The long-term effect of obesity on glaucoma was evaluated using a subgroup analysis (Figure 2). In adults aged 60-70 years, obesity increased the risk of glaucoma by 0.083 compared to participants who were not overweight (Estimate, 0.083; 95% CI, 0.032 to 0.134). In participants who were single, obesity was associated with a 0.075 higher glaucoma incidence (Estimate, 0.075; 95% CI, 0.048 to 0.101). In rural residents and smokers, obesity reduced the risk of glaucoma by 0.085 (Estimate, -0.085; 95% CI, -0.107 to -0.062) and 0.031 (Estimate, -0.031; 95% CI, -0.056 to -0.007), respectively.

Obesity reduced the risk of glaucoma to a certain extent, regardless of diabetes, hypertension, or dyslipidemia diagnosis. However, obesity reduced the risk of glaucoma even more in participants with diabetes, hypertension, or dyslipidemia than in healthy participants. In participants with diabetes, obesity was associated with a reduction of 0.059 (Estimate, -0.059; 95% CI, -0.087 to -0.029) in glaucoma risk, whereas in participants without diabetes, obesity was associated with a reduction of 0.052 in glaucoma risk (Estimate, -0.052; 95% CI, -0.082 to -0.023). Similarly, in individuals with and without hypertension, obesity reduced the risk of glaucoma by 0.039 (Estimate, -0.039; 95% CI, -0.059 to -0.020) and 0.032 (Estimate, -0.032; 95% CI, -0.054 to -0.009), respectively. In participants with and without dyslipidemia, obesity was associated with a glaucoma risk reduction of 0.048 (Estimate, -0.048; 95% CI, -0.070 to -0.025) and 0.029 (Estimate, -0.029; 95% CI, -0.049 to -0.008), respectively, compared to participants who were not overweight. In addition, the risk of glaucoma was 0.065 lower in obese participants with kidney disease than in participants with kidney disease who were not overweight (Estimate, -0.065; 95% CI, -0.110 to -0.020).

## DISCUSSION

This cohort study found that obesity was associated with a decreased risk of glaucoma. After adjusting for demographic, socioeconomic, and health-related variables, we found that obesity reduced the risk of glaucoma by 10.2% compared to participants who were not overweight. The association between obesity and glaucoma was particularly strong in rural residents and smokers. In addition, obesity reduced the risk of glaucoma regardless of diabetes, hypertension, or dyslipidemia diagnosis.

Previous studies have reported associations between obesity and glaucoma in older individuals, but with inconsistent results. A study based using data from the KNHANES found that being overweight (BMI≥ 25.0 kg/m^2^) reduced the risk of POAG (OR, 0.78; 95% CI, 0.63 to 0.96) compared to participants 40 years and older who were not overweight (BMI < 25.0 kg/m^2^) [3]. Another prospective cohort study of 78,777 females in the Nurses’ Health Study and 41,352 males in the Health Professionals Follow-Up Study found no association between cumulatively averaged BMI and POAG subtypes [6]. A study of a large Japanese population conducted cross-sectional and longitudinal analyses and concluded that obesity was an independent risk factor for increased intraocular pressure (IOP) [9].

The present study used both multivariate logistic regression analysis and a Cox proportional hazards model to examine the long-term effects of obesity on glaucoma. Glaucoma was not associated with obesity in model 1, but model 2 showed a significant association in the Cox proportional hazards model. It may be that the association between obesity and glaucoma was affected by metabolic diseases (diabetes, hypertension and dyslipidemia) and interactions among independent variables in model 1. Obesity had a significant effect on glaucoma after adjustment for metabolic diseases in model 2 and in the analysis of the mixed-effects model. In accordance with previous studies, we found that obesity was associated with significantly reduced glaucoma risk in Chinese participants 45 years of age or older. Multivariate logistic regression analysis showed an 11.8% reduction in risk compared to non-overweight individuals, while the Cox proportional hazards model showed a 10.2% reduction in glaucoma risk.

Several mechanisms may explain the association between obesity and glaucoma risk. First, BMI has been shown to be linearly correlated with orbital cerebrospinal fluid pressure (CSF-P) [8]. Lower CSF-P has been investigated as a possible risk factor in the pathogenesis of glaucoma [8]. It is possible that an increase in CSF-P may be caused by hypoventilation or obstructive sleep apnea, leading to respiratory acidosis, which is more common in people with a high BMI [10]. Second, deficiencies in certain nutrients such as glutathione, nitric oxide, and flavonoids, have been found to increase the risk of glaucoma [11,12]. People with low BMIs are more likely to lack specific nutrients [13]. Therefore, we speculate that people with low BMIs are at increased risk of glaucoma due to a lack of nutrients. In addition, since China is a developing country, obesity is, to some extent, associated with a higher standard of living. Individuals who are obese have solved the basic problems of food and clothing and may be able to focus on their health. As a result, they may be more likely to receive screenings for high IOP during routine physical examinations and early interventions to prevent progression to glaucoma. In particular, glaucoma status in this study was self-reported, which might be particularly biased by access to medical care and level of medical understanding.

Our stratified analysis showed stronger associations between obesity and an increased risk of glaucoma in participants aged 60-70 years and in single individuals. In China, single people may face social difficulties and therefore be unwilling to go to public places such as hospitals [14,15]. This could delay an early diagnosis of high IOP and lead to the development of glaucoma. Among people living in rural areas, we found an association between obesity and a decreased risk of glaucoma. We speculate that, as living standards are generally poor in rural China, obese individuals in rural regions have a higher standard of living. Unlike rural residents who are not overweight, they may have sufficient money to detect and control high IOP at an early stage to prevent glaucoma. We also found that, among smokers, obesity was associated with a decreased risk of glaucoma. There is some uncertainty about the association between smoking and glaucoma. A previous study found that maternal smoking and household smoking increased the risk of children being overweight or obese [16]. A cross-sectional study using data from the Global School-Based Student Health Survey found that adolescents who reported daily secondhand smoking were significantly more likely to be obese [17]. Thus, we speculated that smoking increased the risk of obesity, which exerted a protective effect against glaucoma.

In individuals with diabetes, hypertension, or dyslipidemia, obesity reduced glaucoma risk more than in healthy people. The relationship between diabetes and glaucoma remains a matter of debate. The larger volume of cerebrospinal fluid (CSF) in patients with diabetes than in healthy individuals may result in increased CSF pressure, which reduces the risk of glaucoma [18]. Obesity and excessive visceral fat distribution are accompanied by alterations in the hypertensive state [19]. Furthermore, in patients with hypertension, CSF flow is decreased, which could contribute to a decreased risk of glaucoma [20]. Dyslipidemia, which is a risk factor for endothelial dysfunction, is associated with glaucoma [21]. Unesterified cholesterol regulates the fluidity, permeability, and electrical properties of cell membranes. Consequently, cholesterol oversupply caused by diet or heredity contributes to neurodegenerative diseases and vision loss, such as glaucoma [22]. Some evidence suggests that treatment for hyperlipidemia, which may include statins or other cholesterol-lowering drugs, may reduce the risk of glaucoma [23,24]. Obese patients with hyperlipidemia are more likely to receive treatment for hyperlipidemia, thereby reducing their risk of glaucoma. A study showed that those who were characterized as metabolically unhealthy non-obese showed a higher risk of developing POAG than those who were metabolically healthy non-obese, but those who were metabolically healthy obese did not, indicating that metabolic status was more important than obesity [25]. Based on the inextricable links of obesity with diabetes and hypertension, more research is needed to confirm the association between obesity and glaucoma.

The strengths of the present study include the large size of the cohort and the population-based retrospective design. Our retrospective cohort study not only found an association between obesity and glaucoma, but also revealed a possible causal relationship. In addition, 2 different statistical methods were used to analyze the association between obesity and glaucoma to ensure the reliability of the results. Moreover, our analysis accounted for the influence of multiple confounders, including socioeconomic status, lifestyle, and health-related disorders.

This study also has some limitations. First, glaucoma was assessed via self-reporting, which inevitably involves a risk of recall bias. Second, we were not able to assess the association between obesity and glaucoma with further stratification by BMI values. Furthermore, although our analysis included many covariates, some confounders were not taken into account, such as diets. Moreover, the associations of obesity with different types of glaucoma were not examined in this study.

In conclusion, we found that obesity was associated with a decreased risk of glaucoma, especially in rural residents, smokers, and people with kidney disease. Obesity also exerted a stronger protective effect against glaucoma in people with diabetes, hypertension, or dyslipidemia than in healthy people. Our study provides new suggestions for the management of glaucoma patients and obese patients. For glaucoma patients, the requirements for weight control can be appropriately relaxed; for obese patients, weight control standards need to be adjusted according to the conditions of eye diseases.

## DATA AVAILABILITY

All methods were carried out in accordance with relevant guidelines and regulations, and all data are available online at the CHARLS project website (http://charls.pku.edu.cn/).

## Figures and Tables

**Table 1.** Basic characteristics of the study population according to total body mass index (BMI) quartiles

Characteristics		Total BMI quartiles, range (median), kg/m^2^				p-value
		Quartile 1, <20.8 (19.4)	Quartile 2, 20.8-23.1 (22.0)	Quartile 3, 23.1-25.7 (24.3)	Quartile 4, >25.7 (27.7)	
No. of participants		3,345	3,328	3,320	3,364	
Sex (female)		45.0	48.9	56.1	62.7	<0.001
Age (yr)		62.1±10.4	59.0±9.8	58.1±9.4	57.1±8.9	<0.001
Education						<0.001
	Primary or below	77.1	70.6	64.1	63.6	
	Middle school	15.7	19.8	22.8	23.4	
	High school	5.1	6.3	8.5	8.1	
	College or above	2.1	3.3	4.6	5.0	
Married or partnered		82.3	86.0	88.5	91.1	<0.001
Urban		28.6	35.4	42.2	46.8	<0.001
Smoking		50.1	42.7	35.8	29.2	<0.001
Drinking						<0.001
	Drink more than once a month	27.8	28.0	23.4	19.5	
	Drink once a month or less	7.4	8.2	8.5	7.4	
	None	64.8	63.8	68.1	73.1	
Diabetes		8.9	11.2	14.2	19.8	<0.001
Hypertension		28.9	34.4	39.7	54.7	<0.001
Dyslipidemia		20.7	28.4	37.3	49.0	<0.001
Kidney disease		7.0	6.4	5.8	6.1	0.096

Values are presented as % or mean±standard deviation.

**Table 2.** Association of total body mass index quartiles with confounders at baseline by multivariate logistic regression analysis

Variables		OR (95% CI)	p-value
Sex (male)		1.24 (1.10, 1.40)	<0.001
Age (yr)			<0.001
	<50	1.00 (reference)	
	50-60	0.99 (0.87, 1.12)	0.822
	60-70	1.31 (1.14, 1.50)	<0.001
	>70	3.25 (2.79, 3.79)	<0.001
Obesity		0.88 (0.80, 0.97)	0.008
Education			0.238
	Primary or below	1.00 (reference)	
	Middle school	1.01 (0.90, 1.13)	0.875
	High school	0.88 (0.73, 1.06)	0.185
	College or above	1.16 (0.94, 1.44)	0.164
Married or partnered		0.65 (0.58, 0.74)	<0.001
Urban		1.60 (1.46, 1.75)	<0.001
Smoking		1.16 (1.03, 1.30)	0.011
Drinking			0.020
	Drink more than once a month	1.00 (reference)	
	Drink once a month or less	0.98 (0.82, 1.17)	0.814
	None	1.15 (1.03, 1.23)	0.014
Diabetes		1.38 (1.23, 1.56)	<0.001
Hypertension		1.25 (1.14, 1.37)	<0.001
Dyslipidemia		0.79 (0.72, 0.87)	<0.001
Kidney		1.20 (1.01, 1.41)	0.036
Constant		0.19	<0.001

OR, odds ratio; CI, confidence interval.

**Table 3.** Association of total body mass index quartiles with confounders at baseline according to a Cox proportional hazards model

Variables		Model 1	p-value	Model 2	p-value
Sex (male)		1.18 (1.09, 1.27)	<0.001	1.18 (1.07, 1.31)	0.001
Age (yr)					
	<50	1.00 (reference)		1.00 (reference)	
	50-60	1.02 (0.91, 1.15)	0.669	0.98 (0.88, 1.10)	0.783
	60-70	1.38 (1.23, 1.55)	<0.001	1.24 (1.09, 1.40)	0.001
	>70	3.05 (2.72, 3.42)	<0.001	2.43 (2.13, 2.76)	<0.001
Obesity		0.95 (0.88, 1.02)	0.190	0.90 (0.83, 0.97)	0.009
Education					
	Primary or below	-		1.00 (reference)	
	Middle school	-		1.01 (0.91, 1.11)	0.890
	High school	-		0.90 (0.76, 1.07)	0.227
	College or above	-		1.11 (0.93, 1.32)	0.236
Married or partnered		-		0.73 (0.66, 0.80)	<0.001
Urban		-		1.45 (1.34, 1.56)	<0.001
Smoking		-		1.15 (1.04, 1.26)	0.006
Drinking		-			
	Drink more than once a month	-		1.00 (reference)	
	Drink once a month or less	-		0.99 (0.85, 1.16)	0.920
	None	-		1.12 (1.02, 1.23)	0.019
Diabetes		-		1.28 (1.16, 1.42)	<0.001
Hypertension		-		1.21 (1.12, 1.30)	<0.001
Dyslipidemia		-		0.83 (0.76, 0.90)	<0.001
Kidney		-		1.14 (0.99, 1.31)	0.061

Values are presented as hazard ratio (95% confidence interval).

**Table 4.** Longitudinal associations of obesity with glaucoma in subgroups according to a mixed-effects model

Variables		Estimate (95% CI)	p-value
Age <50 * Obesity		0.054 (-0.002, 0.111)	0.058
	Age 50-60 * Obesity	0.039 (-0.011, 0.089)	0.123
	Age 60-70 * Obesity	0.083 (0.032, 0.134)	0.001
	Age >70 * Obesity	-0.039 (-0.102, 0.023)	0.214
Married or partnered * Obesity		-0.058 (-0.120, 0.004)	0.067
	Others * Obesity	0.075 (0.048, 0.101)	<0.001
Urban * Obesity		-0.073 (-0.094, 0.015)	0.158
	Rural * Obesity	-0.085 (-0.107, -0.062)	<0.001
Smoking * Obesity		-0.031 (-0.056, -0.007)	0.012
	Non-smoking * Obesity	-0.016 (-0.047, 0.015)	0.312
Diabetes * Obesity		-0.059 (-0.087, -0.029)	<0.001
	Without diabetes * Obesity	-0.052 (-0.082, -0.023)	<0.001
Hypertension * Obesity		-0.039 (-0.059, -0.020)	<0.001
	Without hypertension * Obesity	-0.032 (-0.054, -0.009)	0.005
Dyslipidemia * Obesity		-0.048 (-0.070, -0.025)	<0.001
	Without dyslipidemia * Obesity	-0.029 (-0.049, -0.008)	0.006
Kidney disease * Obesity		-0.065 (-0.110, -0.020)	0.005
	Without kidney disease * Obesity	-0.009 (-0.045, 0.026)	0.605

CI, confidence interval.
